# No Major Age Shift During the Second Wave of COVID-19: A Mortality Analysis at a Tertiary-Level Hospital

**DOI:** 10.5152/TJAR.2023.22815

**Published:** 2023-02-01

**Authors:** Kelika Prakash, Venkata Ganesh, Arshad Ayub, Richa Aggarwal, Yudhyavir Singh, Shailendra Kumar, Abhishek Singh, Kapil D. Soni

**Affiliations:** 1 Department of Anaesthesiology, Pain Medicine and Critical Care, All India Institute of Medical Sciences (AIIMS), New Delhi, India

**Keywords:** COVID-19, mortality, pandemic, second wave

## Abstract

**Objective:**

The second wave of coronavirus epidemic affected India severely. We reviewed the in-hospital deaths during the second wave at a dedicated COVID hospital to better understand the clinical characteristics of patients who died during this period.

**Methods:**

Clinical charts of all patients who were admitted and died in-hospital due to COVID-19 between 1 April 2021 and 15 May 2021 were reviewed and clinical data were analysed.

**Results:**

The total number of patients admitted to hospital and the intensive care unit was 1438 and 306, respectively. The in-hospital and intensive care unit mortality was 9.3% (134 out of 1438 patients) and 37.6% (115 out of 306 patients), respectively. Septic shock with multiorgan failure was the cause of death in 56.6% of the deceased patients (n = 73) and acute respiratory distress syndrome in 35.3% (n = 47) patients. Of the deceased, 1 patient was less than 12 years old, 56.8% were between 13 and 64 years of age and 42.5% were geriatric, that is, 65 years of age or older. There were no comorbidities in 35.1% of the deceased patients. The cause of death did not vary with the age group.

**Conclusion:**

The in-hospital and intensive care unit mortality during the second wave was 9.3% and 37.6%, respectively. There was no major age group shift in the second wave as compared to the first wave. However, a significant number of patients (35.1%) did not have any comorbidity. Septic shock with multiorgan failure was the most common cause of death followed by acute respiratory distress syndrome.

**Main Points**

- The in-hospital and intensive care unit mortality was 9.3% and 37.6%, respectively.
- Septic shock with multiorgan failure was the most common cause of death.
- Of the deceased patients, 42.5% were geriatric, that is, 65 years of age or older.
- Of the deceased patients, 35.1% did not have any comorbidity.

## Introduction

The second wave of the coronavirus epidemic overwhelmed the health system of India. The number of patients affected per day and the number of deaths per day were one of the highest in the world (www.worldometers.info/coronavirus/country/india, https://www.covid19india.org/). Lack of adequate vaccinations, prominence of variants of SARS-COVID-19 virus, and absence of coronavirus-appropriate social protocols were some of the reasons cited for the overwhelming number of cases. There is a public perception that the second wave had a higher mortality and that the proportion of young patients who died during the second wave was higher than that during the first wave. We investigated the demographic and clinical profile of patients who died due to COVID-19 during the second wave at tertiary care hospital from 1 April 2021 to 15 May 2021.

## Methods

We conducted a retrospective observational study and reviewed charts of all patients who were admitted and died in a tertiary COVID care centre in New Delhi, India during the second wave of COVID-19 pandemic affecting India from 1 April 2021 to 15 May 2021. Data was analysed as a subgroup of an ongoing study following institutional ethical clearance from the institutional ethics committee of All India Institute of Medical Sciences, New Delhi, India (IEC-291/17.4.2020). As this study was a retrospective study on deceased patients, informed consent was not feasible. We evaluated demographic and clinical characteristics including comorbidities from clinical charts. The duration of illness and treatment received during the course of intensive care unit (ICU) stay was reviewed. The cause of death was identified from the death certificates of the deceased. The proportion of geriatric patients, that is, those 65 years of age or older was noted, and the cause of death and the presence of comorbidities were compared between geriatric and non-geriatric groups.

All patients were treated and managed according to the institutional protocol which is briefly described. Oxygen therapy was provided to a target SpO_2_ of 88% to 95% using face masks, non-rebreathing masks, high flow nasal cannula (HFNC), non-invasive ventilation (NIV) as necessary. All hypoxic patients were advised self/awake proning and were prescribed methyl-prednisolone (1-2 mg kg^−1^) and remdesivir. The acute respiratory distress syndrome (ARDS) protocol was followed for management of intubated patients which included lung protective ventilation and proning when the P/F ratio (which is the arterial pO_2_ from the arterial blood gas divided by the FiO_2_ or the fraction (per cent) of inspired oxygen that the patient is receiving) was less than 150.^1,2^ Patients in ICU were given broad-spectrum antibiotics which was then modified once cultures were available. Enoxaparin or dalteparin was administered for thromboprophylaxis. Other investigations and supportive management with fluids, nutrition, treatment of comorbidities, and interventions like dialysis were provided as required. The levels of inflammatory markers (interleukin-6, C-reactive protein, and ferritin) and procalcitonin on the day of arrival to ICU were measured. These were then repeated in case of clinical worsening at the discretion of the treating doctor. Patients who developed cytokine storm were treated with 120 mg of methylprednisolone as per institutional protocol.

### Statistical Analysis

Data are presented as mean ± standard deviation or median (minimum-maximum) as applicable. Chi-square test was used to compare categorical variables between 2 groups. Mann–Whitney *U* test was used to compare non-parametrical data. *P* value less than .05 was considered significant. Data were analysed using R statistical software version 4.0.0.

## Results

Total number of patients admitted to the COVID hospital from 1 April 2021 to 15 May 2021 was 1438. One hundred thirty-four patients died during this period. Patients were shifted to ICUs either when the oxygen requirement was more than 10 L min^−1^ or following an acute cardiovascular/cerebral event or any deterioration in clinical condition which required ICU care as determined by the treating physician. Nineteen patients (14.17%) died in the wards before they could be shifted to an ICU. Three hundred six (21.27%) patients were admitted to the ICU, of whom 115 died. The all-cause mortality rate was 9.3% for in-hospital deaths and 37.6% for deaths in the ICU. The characteristics of the deceased patients were analysed.

### Demography and Clinical Characteristics

The age of the adult deceased patients was 61.3 (18-93) years. There was 1 paediatric death, who was a preterm neonate who died at 2 days of age due to congenital cyanotic heart disease and heart failure. Seventy-six patients (56.7%) were between 13 and 64 years of age and 57 patients (42.5%) were geriatric, that is, 65 years of age or older (Table 1). There were more males than females (62.4% vs. 37.6%).

### Presenting Illness

**Figure 1.**
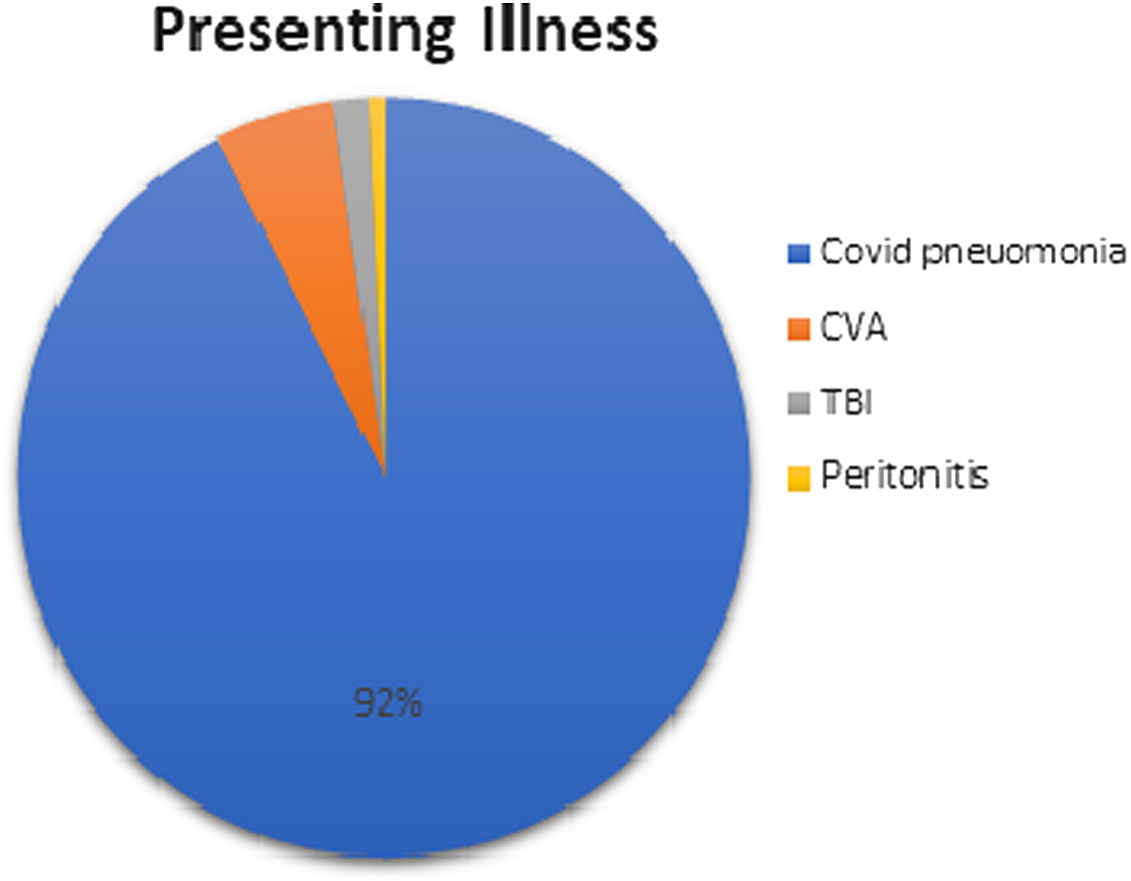
Presenting illness and oxygen requirement. CVA, cerebrovascular accident; TBI, traumatic brain injury.

The majority of the deceased (123 patients, 91.8%) were admitted to the hospital due to COVID pneumonia and hypoxia. Seven patients (5.2%) presented with cerebrovascular accidents (6 ischaemic infarcts and 1 intracranial bleed). Two patients (1.5%) were admitted due to traumatic brain injury. One patient (7.5%) presented with biliary peritonitis following a laparoscopic cholecystectomy (Figure 1).

### Comorbidities

**Figure 2.**
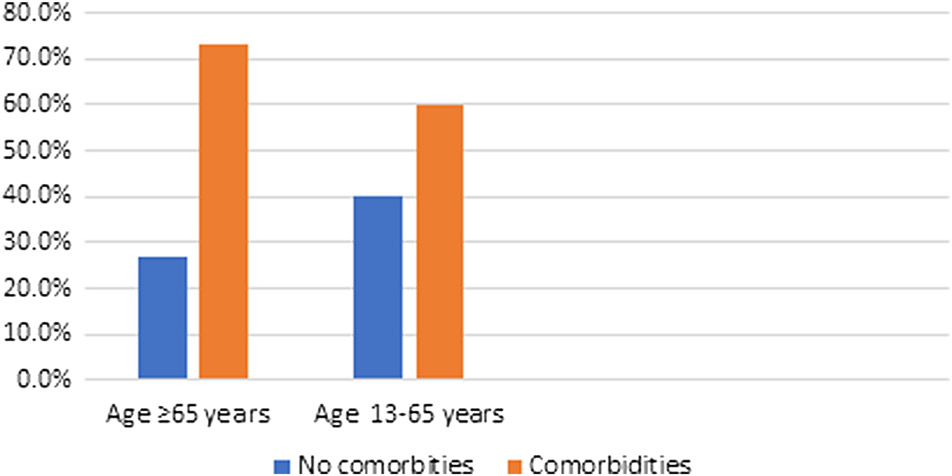
Number of patients with and without comorbidities.

Forty-seven patients (35.1%) had no comorbidities. Of the patients, 21.6% suffered from 1 comorbidity while 43.3% patients had 2 or more comorbidities (Table 1). The most common co-morbidity was hypertension followed by diabetes mellitus (54 patients, 32.8% and 43 patients, 32.1%, respectively). Twenty-three patients had chronic kidney disease (17.1%). Twenty-one patients had a history of coronary artery disease (15.7%). The rest of the patients had chronic liver failure (n = 4), old cerebral infarcts (n = 3), underlying malignancy (n = 5), valvular heart disease (n = 2), autoimmune disease, tubercular meningitis, and hydrocephalus (1 each). Though the number of patients who had comorbidities was higher in geriatric group as compared to non-geriatric group, this difference was not statistically significant (73.2% vs. 60.0% respectively, *P* = .100, Figure 2).

**Figure 3.**
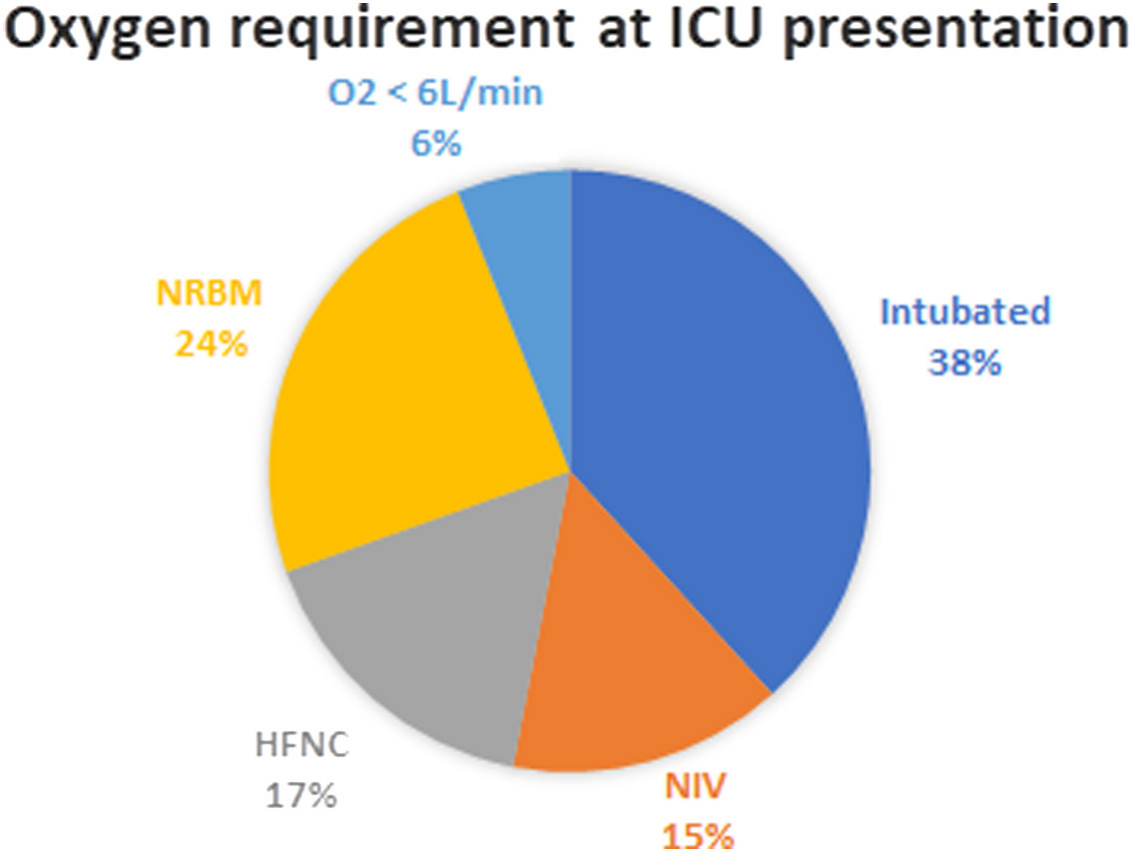
Oxygen requirement at presentation to the ICU. HFNC, high flow nasal cannula; NRBM, non-rebreathing mask; ICU, intensive care unit; NIV, non-invasive ventilation.

Fifty-two per cent of the patients admitted to the ICUs were in-patients, that is, they were transferred to an ICU from a ward, and 48% of the patients were admitted directly to the ICU from the emergency. The oxygen requirement of patients on arrival to the ICU was as follows. Forty-four (38.3%) patients either required intubation on arrival to ICU or were received in the ICU in an intubated condition. Twenty-eight patients (24.4%) needed oxygen at greater than 10 L min^−1^ with a non-rebreathing mask. Seventeen patients (14.8%) required NIV while 19 patients (16.5%) needed HFNC. Seven patients (6.1%) had an oxygen requirement of less than 6 L min^−1^ maintaining oxygenation on a face mask or nasal prongs (Figure 3).

The mean sequential organ failure assessment score (SOFA score) on admission to ICU was 9.3 ± 3.5. The levels of inflammatory markers on the day of arrival to ICU were as follows: interleukin-6: 92.8 (31.5-1620) pg mL^−1^; C-reactive protein 14.7 (9.6-547) mg mL^−1^; ferritin 643 (36-1358) ng mL^−1^, and procalcitonin 0.03 (0.02-97) ng mL^−1^.

The most frequent complication seen was sepsis (n = 81, 60.4%) followed by acute kidney injury (n = 69, 51.5%). Hemodialysis was required in 27 patients (20.1%). Eleven patients had barotrauma and pneumothorax which was managed by insertion of intercostal drains. Four patients developed intracranial bleeds during the course of ICU stay. Cardiogenic shock was seen in 5 patients. The number of days from hospital admission till intubation was 3 days (0-17). During the course of ICU stay, 49 patients (42.2%) developed “cytokine storm.” These patients received 120 mg of methyl prednisolone and tocilizumab.

The duration of ICU and hospital stay was 4 days (1-73) and 9 days (1-76), respectively. The cause of death in majority of the patients was septic shock leading to multiorgan failure (MOF) (n = 75, 56.0%). In 36.6% (n = 49) patients, ARDS was identified as the cause of death. Six patients (4.5%) died of a cardiogenic shock and 4 patients (3.0%) due to raised intracranial pressure (Table 2). Septic shock with MOF followed by ARDS were the most common causes of death in patients immaterial of age group (*P* = .391) or the presence of comorbidities (*P* = .103).

### Comparison Between First and Second Wave Data

The authors have previously reported a mortality analysis from the same centre during the first wave.^3^ Table 3 provides a comparison between the first and the second wave of the pandemic. The ICU mortality rate during the second wave and the first wave was comparable (37.6% vs. 36.1%, *P* = .667) though the in-hospital mortality during the second wave was significantly less than that in the first wave (18.2% vs. 9.3%, *P* < .001). The data of the adult deceased patients were compared. Among the adult deceased patients, a larger percentage of patients were geriatric as compared to the first wave (42.5% patients in the second vs. 25.5% during the first wave, *P* < .001). There was a significantly higher percentage of patients who had no comorbidities in the second wave (35.1% vs. 5.3%, *P* < .001). Septic shock with MOF was the most common cause of death followed by ARDS in both waves of the pandemic.

## Discussion

We conducted a mortality analysis in the second wave of the coronavirus pandemic and found that the overall in-hospital mortality was 9.3% and ICU mortality was 37.6%.

During the first wave, we reported that among those deceased, the percentage of patients more than 65 years of age was 23.1%.^3^ In the second wave, we have now found that among the deceased, 42.5% patients were geriatric, that is, 65 years of age and older. While there is public apprehension that during second wave, younger patients contributed to a greater percentage of deaths than during the first wave, our analysis shows the contrary. The proportion of geriatric patients was higher in the second wave as compared to the first wave (*P* < .001). This is in concurrence with the observations made by Ioannidis et al.^4^ They analysed data from 49 countries and reported that older patients were more severely affected during both waves.^4^ Similarly, in a meta-analysis conducted by Qui et al, the authors found that the median age of patients who died due to COVID-19 from January 1 to April 2020 was 69.9 years.^5^

It has been reported that mortality due to COVID-19 is high in patients with comorbidities and relatively healthier patients were unlikely to die.^3^ In a meta-analysis conducted on studies on COVID-19-related deaths, which were published between January and April 2020, the authors found that 72.21% of deceased patients had underlying diseases.^5^ Imam et al^6^ in their study on 1305 patients admitted to a hospital in the United States reported that increase in age and number of comorbidities were associated with higher mortality. Indeed, in an earlier analysis from the same centre, we reported that 94.74% of all deceased adult patients had at least 1 comorbidity.^3^ The alarming result from the current analysis is that during the second wave, 35.1% of the deceased patients had no comorbidities. This figure is much higher than the first wave where only 5.26% patients had no comorbidities, *P* < .001. It is also interesting to note that the overall in-hospital mortality in the first wave and second wave reported from our centre is 18.2% and 9.3%, respectively.^3^ Though the in-hospital mortality was lower in the second wave as compared to the first wave (*P* < .001), the overall mortality in the ICU was similar (36.1% and 37.6%, *P* = .667).^3^ This was in spite of the fact that there is, arguably, more clarity on treatment and better hospital preparedness during the second wave as compared to the first wave. It is possible that variant strains, especially the B1.617.2 strain, which were prominent during the second wave, are more pathogenic leading to a high number of fatalities.^7^ However, we do not have data on the strain of virus affecting our patients in the ICU during this period and can only presume based on epidemiological prevalence that the B1.617.2 strain was prevalent among our patients too.

Patients with viral pneumonia are susceptible to secondary infections. In our study group, septic shock with MOF was the major cause of mortality both in the second and first waves. This is in concurrence with data from various centres in the world where secondary infection and septic shock was a major killer.^3,8,9^

While there are fears that the second wave resulted in higher death rate, we found that the ICU mortality rate was similar to the first wave and the in-hospital mortality rate was actually lower. We also found that during the second wave of the COVID-19 pandemic, there was no major age shift though the number of patients who did not have any comorbidity was high.

There are limitations to this study. First, as we only analysed charts of patients who died, we cannot comment on the effect of treatment or the differences in clinical characteristics or laboratory parameters among survivors and non-survivors. Secondly, as mentioned previously, we do not have data on the strains of coronavirus at our hospital. Lastly, this study is a single-centre retrospective study and is not representative of a larger population.

## Conclusion

In conclusion, we found that during the second wave of the pandemic, the overall in-hospital and ICU mortality was 9.3% and 37.6%, respectively. Septic shock with MOF followed by ARDS was the most common cause of death. Among the patients who died, the proportion of patients who had no comorbidities was high (35.1%). There was no major shift in age in the second wave.

## Figures and Tables

**Table 1.** Demography and Clinical Characteristics

Age	61.3 (18-93) years
Male: female	83 males: 50 females (62.4%: 37.6%)
SOFA score	9.3 ± 3.5
Number of comorbidities	
Nil	47 (35.1%)
One	29 (21.6%)
Two	30 (22.4%)
Three	20 (14.9%)
≥4	8 (5.9%)
Inflammatory markers	
IL-6	92.8 (31.5- 1620) pg mL^-1^
CRP	14.7 (9.6-547) mg mL^-1^
Ferritin	643 (36- 1358) ng mL^-1^
Procalcitonin	0.03 (0.02- 97) ng mL^-1^
Comorbidities	
No comorbidity	47 (35.1%)
Hypertension	54 (40.2%)
Diabetes mellitus	43 (32.1%)
Chronic kidney disease	23 (17.2%)
Coronary artery disease	21 (15.7%)
Chronic liver disease	4 (2.9%)
Malignancy	5 (3.7%)
Others	8 (5.9%)

Data are represented as mean ± standard deviation, median (minimum-maximum), or number (percentage of total).

CRP, C-reactive protein; IL-6, interleukin-6; SOFA score, sequential organ failure assessment score.

**Table 2.** Clinical Course

ICU mortality	115 (37.6%)
Hospital mortality	134 (9.3%)
Duration of ICU stay	4 days (1-73)
Duration of hospital stay	9 days (1-76)
No of days from admission to intubation	3 days (0-17)
Complications	
Sepsis	69 (51.5%)
AKI	58 (43.3%)
Barotrauma	11 (8.3%)
Intracranial bleed	4 (3.0%)
Cardiogenic shock	5 (3.7%)
Cause of death	
Septic shock with multiorgan failure	75 (56.0%)
ARDS	49 (36.6%)
Cardiogenic shock	6 (4.5%)
Raised intracranial pressure	4 (3.0%)

Data are represented as mean ± standard deviation, median (minimum-maximum), or number (percentage of total).

AKI, acute kidney injury; ARDS, acute respiratory distress syndrome; ICU, intensive care unit.

**Table 3.** Comparison Between First and Second Waves of the Pandemic

	First Wave	Second Wave	*P*
ICU mortality	253/700 (36.1%)	115/306 (37.6%)	.667
Hospital mortality	260/1420 (18.2%)	134/1438 (9.3%)	.001^#^
Age distribution*			
18 to 65 years	184 (74.5%)	76 (56.7%)	.001^#^
≥65 years	63 (25.5%)	57 (42.5%)	
Gender (male : female)*	163 : 84	83 : 50	.481
Comorbidities*			
Yes	234 (94.7%)	86 (64.9%)	.001^#^
No	13 (5.3%)	47 (35.1%)	
Cause of death*			
Septic shock with multiorgan failure	136 (55.1%)	75 (56.0%)	.002^#^
Severe ARDS	63 (25.5%)	49 (36.6%)	
Others	48 (19.4%)	10 (7.4%)	

First wave: 4 April 2020 to 4 July 2020; second wave: April 1 2021 to 15 May 2021. Data are represented as number (percentage of total).

*Data of adult deceased patients which was 247 in the first wave and 133 in the second wave.

^#^ *P* value is significant.
